# Toothbrushing Frequency in Saudi Arabia: Associations with Sociodemographics, Oral Health Access, General Health, and Diet

**DOI:** 10.3390/ijerph22050764

**Published:** 2025-05-13

**Authors:** Naif Nabel Abogazalah, Amani Alzubaidi, Saleh Ali Alqahtani, Nada Ahmad Alamoudi, Esperanza Angeles Martinez-Mier

**Affiliations:** 1 Department of Restorative Dental Sciences, King Faisal University College of Dentistry, Hufof 36362, Saudi Arabia; ssofan@kku.edu.sa (S.A.A.); naalamoudi@kku.edu.sa (N.A.A.); 2 Department of Maxillofacial Dental Surgery, King Khalid University College of Dentistry, Abha 61421, Saudi Arabia; amalzubaidi@kku.edu.sa; 3 Department of Dental Public Health and Dental Informatics, Indiana University School of Dentistry, Indianapolis, IN 46202, USA; esmartin@iu.edu

**Keywords:** toothbrushing frequency, oral hygiene, dental hygiene, preventive dentistry

## Abstract

This study explores the toothbrushing frequency and its association with sociodemographic factors, health status, and dietary habits in Saudi Arabia. Using data from the 2017 National Demographic and Health Survey by the Ministry of Health, we analyzed responses from 44,779 individuals aged five and older. Statistical analysis using SPSS and multinomial regression revealed that 57.3% of the population brushed their teeth less than once a day. Differences were noted across regions, ages, and genders. Key factors associated with increased brushing frequency included age (45–54 vs. older than 60), nationality (Saudi vs. non-Saudi), region (Western vs. Central), and marital status (married vs. non-married). Conversely, individuals with co-morbidities, disabilities, smokers, and those without prior dental treatment were less likely to maintain recommended oral hygiene practices. Our findings suggest that toothbrushing practices fall short of professional recommendations, highlighting a need for enhanced educational efforts. Oral health care providers in Saudi Arabia are encouraged to implement regular awareness programs to improve brushing habits and overall oral hygiene.

## 1. Introduction

Toothbrushing is a fundamental oral hygiene practice involving the mechanical removal of dental plaque and debris from the teeth and gums using a toothbrush and toothpaste. This routine procedure is crucial not only for maintaining oral health but also for preventing a range of dental diseases, such as dental caries and periodontal disease [1].

Dental caries and periodontal diseases are highly prevalent and remain a major health problem worldwide [2]. The major risk factor for both dental caries and periodontal diseases is the accumulation of dental plaque (biofilm) on tooth surfaces [3]. Dental caries develops when there is an imbalance between protective and pathogenic factors in the dental biofilm, as cariogenic bacteria (within the dental biofilm) consume fermentable carbohydrates to produce acids that cause enamel demineralization. The reversal of this demineralization is influenced by salivary flow and composition, fluoride exposure mainly from toothpaste, and oral hygiene practices [4]. Regarding periodontal diseases, they primarily arise from the accumulation of dental plaque, which harbors pathogenic bacteria, leading to an inflammatory response in the periodontal tissues. When dental plaque is not removed for ten to twenty days, it can lead to the development of gingivitis, which may progress to periodontitis, resulting in bone loss and ultimately tooth loss [5,6,7].

According to recent global estimates, 621 million children had untreated dental caries in primary teeth, while 2.4 billion people have untreated dental caries affecting their permanent teeth [8]. Severe periodontitis is reported to affect approximately 743 million people worldwide [9]. The prevalence of dental caries in Saudi Arabia (SA) has been increasing due to social, economic, and environmental changes that started in the seventh decade of the last century. Recent meta-analysis showed that the average prevalence of dental caries in the primary dentition was 75.43% while in permanent dentition was 67.7% [10]. Regarding periodontal diseases, periodontitis affected 8.6% of high school students in SA, with higher rates among those not regularly brushing their teeth or visiting dental clinics regularly [11].

While professional recommendations advocate brushing twice a day, variations in toothbrushing habits have been observed across different populations globally. A recent systematic review [12] indicated varying toothbrushing frequencies across different global regions. Based on World Health Organization’s classification of world regions, the review found that the Americas and the Western Pacific regions demonstrated strong oral hygiene practices, with 82.9% and 81.4% of their populations, respectively, brushing twice or more daily. Similarly, the South-East Asian Region showed a high prevalence of frequent brushing at 77.1%. In contrast, the Eastern Mediterranean Region, which includes SA, only 41.4% of participants brush their teeth twice or more daily, while a significant 32.1% rarely or never brush (data from SA itself was not included in this review) [12].

In SA, data from El Bcheraoui et al. (2013) [13] revealed that a notable 16.3% of SA’s population have never brushed their teeth, with 71.5% brushing at least once daily. However, the study did not specify the percentage brushing twice or more daily, nor does it explore determinants influencing brushing frequency. This comparison underscores the need for further research in SA to align with global efforts and improve national oral hygiene standards.

This paper aims to explore the toothbrushing frequency and its association with sociodemographic factors, general health, access to oral health care, and diet-related variables at the national level using data from the National Demographic and Health Survey (NDHS) conducted by SA’s Ministry of Health (MOH) in 2017.

## 2. Materials and Methods

The data source was the National Demographic and Health Survey (NDHS) conducted by SA’s Ministry of Health (MOH) in 2017 (Central IRB log No: 20—204E). The data were available from the office of Directorate of Primary Health Care Centers (MOH headquarters, Riyadh, Saudi Arabia). Details about survey design and sampling procedure have been published previously [14]. Briefly, the MOH used a probability multistage stratified random sampling for the NDHS. Prepared house to house visits were conducted to interview head of a family or an eligible representative and other specific family member between 12 February 2017, to 23 May 2017. Interviews were conducted with 45,287 household heads: 40,955 adult and 15,585 children family members. The participants answered questions related to demographics, education, family income, environmental sanitation, mortality, morbidity, nutrition, tobacco use, oral health, and health services utilization. Eligible datasets were those with complete relevant sociodemographic responses to oral health questions.

### 2.1. Variables

The dependent variable was daily toothbrushing frequency as less than once, once, and twice or more per day as participants responded to the question, “How many times do you wash your teeth with a brush and toothpaste during the last 30 days?” with a range of options: “I have never cleaned my teeth”, “I clean my teeth some days but not daily”, “Once weekly”, “Many times per week”, “Once daily”, and “Twice or more daily”. Independent variables included the following (Table 1): sociodemographic variables: age group, gender, nationality, geographic region, marital status for household head, completed education level, total monthly income, residence crowding. General health variables: relevant medical conditions, history of physical accident, history of physical disability, smoking, body mass index (BMI), household head health insurance; oral health-related variables: dental health care availability in the last year, dental visits frequency in the past year, reasons for last dental visit, regular source of dental care; diet-related variables: frequency of consuming sweets, frequency of drinking soft drinks.

### 2.2. Data Analyses

We selected the variables based on availability from the entire NDHS-2017 dataset, and based on a comprehensive review of existing literature, theoretical frameworks, and their relevance to toothbrushing behavior. The data were collected and statistically analyzed using SPSS version 20.0 software. For categorical variables, frequency distributions and relative frequencies were calculated. Cross tabulation was performed to express the categorical variables and Chi-square test was used to perform the bivariable analysis. Multinomial logistic regression analysis was performed to meet our aim of assessing the associations between toothbrushing frequency and independent variables described above among SA residents. Odd ratios and confidence intervals were reported. Analysis was performed with consideration for sample weights to provide estimates representative for SA residents [14].

## 3. Results

The present study included responses from 44,779 subjects, 54% females, 90% were Saudis from Western (31%), Central (21%), Eastern (20%), Southern (19%), and Northern (9%) regions, out of which 25,954 reported brushing less than once daily; 11,788 subjects brushed their teeth once daily, and 7037 brushed twice or more than twice a day (Table 1).

The bivariable analysis results can be found in Table 1. For respondents of ages 5–14 years old, 62.4% brushed their teeth less than once a day. For those 65 years of age or older, 30.9% and 17.7% brushed their teeth once a day or more than twice a day, respectively. Most males (58.6%) brushed less than once a day, whereas a greater number of females brushed either once or twice a day. Those who brushed less than once a day, were mainly from North region, whereas those who brushed once a day mainly resided in the West region, while most of those who brushed twice a day resided in the Central region. Those who were with primary level of education mainly brushes less than once daily (60.3%). It was observed that more respondents with low monthly income brushed less than once a day than those with higher monthly income. Subjects who reported co-morbidities, were mostly brushing less than once a day. Smoking was more prevalent in respondents who either brush less than once or once daily. Those who brushes less than once a day, had health insurance (60.5%) for themselves and for their family members. Most of the respondents who brushed less than once daily (58.2%) had no available dental care when needed in the past year. Interestingly, those who brushed less than once daily, also did not report eating sweets (65.7%) (Table 1).

Multinomial regression results can be found in (Table 2). The age group of 45–54 years old had an increased likelihood of brushing twice or more daily, over 5 times more, than the age group of +65 (OR = 5.444, 95%CI = 1.105–26.826). The odds for toothbrushing once daily vs. less than once daily for males were 39.5% less than for females (OR = 0.605, 95%CI = 0.403–0.907). The Western region of SA showed an increased likelihood of brushing twice or more daily, approximately 9 times more than the Central region (OR = 9.159, 95%CI = 4.110–20.414). Those who were married showed an increased likelihood of brushing twice or more daily—3 times more than the non-married group (OR = 3.620, 95%CI = 1.076–12.175). The likelihood of smokers brushing their teeth twice or more daily was higher than that of nonsmokers (OR = 3.382, 95%CI = 1.192–9.600). The group of people who were not eating sweets at all showed a decreased likelihood of brushing their teeth twice or more daily than people who consume sweets at least once daily by 76.9% (OR = 0.231, 95%CI = 0.081–0.653). The group of people who drank soft drinks many times per week, reported brushing their teeth twice or more daily—approximately 3 times more than the people who drink soft drinks at least once daily (OR = 3.331, 95%CI = 1.131–9.811).

## 4. Discussion

Oral diseases can be prevented through proper oral hygiene practices such as frequent toothbrushing with fluoridated toothpaste [1]. In this study, we investigated the patterns of toothbrushing frequency among residents of Saudi Arabia (SA) and their association with sociodemographic, general health, and diet-related factors. Our analysis revealed significant variations in toothbrushing habits across different age groups, genders, and regions.

Notably, only 15.8% of the population reported brushing their teeth twice or more daily, aligning with professional dental recommendations [1]. A substantial portion of the population, particularly males and those with lower educational levels, reported brushing their teeth less than once a day. Individuals residing in the Northern region (the least populous and developed), those with lower income, and those without regular access to dental care were more likely to brush less frequently. In contrast, younger adults, females, and those from the Western region (the most populous and more developed) were more likely to brush twice or more daily.

Our study revealed similarities when compared to the earlier work by El Bcheraoui et al. (2013) [13]. They reported that 71.5% of Saudi Arabian individuals aged 15 and above brushed their teeth at least once daily, which is relatively consistent with our finding that a large portion of the population brushes at least once per day. However, our study shows that only 15.8% adhere to the recommended practice of brushing twice or more daily, while it was not reported in El Bcheraoui et al. (2013) [13]. Moreover, both studies agree on the positive impact of higher educational levels on dental clinic visits and routines, suggesting that education remains a pivotal factor in promoting better oral hygiene behaviors. Additionally, gender differences identified in both studies underscore the need for targeted interventions, as males were generally less likely to engage in regular toothbrushing.

The results from our study indicated that toothbrushing frequency in SA is concerning, with 57.3% of participants brushing less than once a day, 26.9% brushing once daily, and only 15.8% brushing twice or more daily, a figure considerably lower than that observed in other countries. The data from the systematic review by Gupta et al. (2024) [12] which investigated toothbrushing frequencies across various global regions revealed significantly higher rates than SA of frequent brushing. For example, in the South-East Asian Region, 77.1% reported brushing twice or more daily, while the Western Pacific Region showed similar results at 81.4%. Even in the Eastern Mediterranean Region, where oral hygiene practices are lower than the other regions, 41.4% of participants still brush twice or more daily compared to only 15.8% in SA. This discrepancy is further highlighted when examining the Gross Domestic Product (GDP)-related findings. Despite SA being classified among the highest GDP countries, where you would expect better oral hygiene practices, the contrast is present. The systematic review [12] also shows that in high GDP countries, like those in the 4th Quintile, 78.6% of population brushes twice or more daily. This is significantly lower than the 15.8% observed in SA. These results suggest that in SA, factors beyond economic status, such as cultural perceptions of oral health or variations in public health initiatives, may heavily influence brushing habits.

Interestingly, behavioral factors such as smoking were associated with increased toothbrushing frequency and people who reported not eating sweets brush their teeth less frequently than those who consume sweets at least once daily, highlighting complex interplays between health behaviors. Studies indicated that smokers are more likely to experience oral health problems, such as halitosis and periodontal diseases, which can motivate them to adopt more rigorous toothbrushing practices as a preventive measure [15,16]. Smokers might brush their teeth more frequently to counteract bad breath and maintain a more socially acceptable image [17,18]. Moreover, the observation that individuals who do not consume sweets tend to brush their teeth less often was unexpected. One reason might be the perception that less sugar intake reduces the need for strict oral hygiene, leading individuals to underestimate the importance of regular brushing. This perception could result in less frequent brushing among those who follow a healthier diet. Additionally, there may be a reporting bias, where individuals who consider their diet healthy might unintentionally underreport their brushing frequency, believing their diet alone is sufficient to maintain oral health. Future research could investigate these factors to better understand the relationship between diet and oral hygiene practices.

Multiple studies have demonstrated a strong inverse relationship between the frequency of toothbrushing and the prevalence of dental caries among adults. A meta-analysis has shown that self-reported infrequent brushers have a significantly higher incidence of dental caries lesions compared to those who brush frequently, with an odds ratio (OR) of 1.50 (95%CI, 1.34 to 1.69) [19]. Another meta-analysis of fourteen studies found that infrequent brushing significantly increases the odds of poor periodontal health (OR 1.41, 95%CI: 1.25–1.58, *p* < 0.0001). However, a lack of studies on the link between brushing frequency and periodontitis is also noted [20].

When examining our findings, it is important to consider cultural and behavioral factors that may affect toothbrushing habits, such as parental influence and media exposure [21]. Although our study relied on data from the 2017 National Demographic and Health Survey, these specific variables were not available in the dataset. We recognize that factors like parental guidance can significantly shape children’s brushing routines, while media campaigns can influence the general public views and practices related to oral hygiene. Addressing these variables could provide a more comprehensive understanding of oral hygiene behaviors in SA. We suggest that future research should explore these influences to better assess their impact on toothbrushing habits in SA.

The use of the 2017 National Demographic and Health Survey provides valuable insights into toothbrushing habits, but it also comes with significant limitations due to the data’s age. Since 2017, societal changes, particularly those resulting from the COVID-19 pandemic, may have impacted health behaviors, access to dental care, and public awareness of oral health. Therefore, the findings should be interpreted with caution, as toothbrushing patterns may have shifted since then. The current landscape of public health challenges and economic conditions may differ markedly from those present in 2017. Moving forward, we recommend that future studies incorporate more recent data to more accurately reflect the state of oral health behaviors in SA and to account for the evolving nature of health practices. Based on our knowledge, the 2017 survey is the most recent publicly available data.

To reduce discrepancies and inequalities in toothbrushing frequency among different demographic groups in SA, targeted interventions are necessary. For older adults over 65, educational programs that emphasize the importance of toothbrushing and flossing through the Saudi Ministry of Health’s (MOH) home medicine services [22]. Moreover, culturally sensitive campaigns tailored for both Saudi and non-Saudi populations can enhance engagement and awareness about the importance of toothbrushing, particularly in various regions such as Western and Central Saudi Arabia. Drawing from successful examples, such as the implementation of teledentistry in the United States [23], which has improved access and quality of care for vulnerable populations like children and rural patients, the SA’s MOH can adopt similar approaches to improve toothbrushing frequency through the already successfully available virtual clinics at the Saudi MOH [24]. Teledenistry through SA’s MOH virtual clinics services can facilitate remote consultations, triage, and follow-up care, increasing access to oral health services, especially in underserved areas [25]. Furthermore, in the Caribbean Island of Anguilla, the value of forming collaborative partnerships with medical schools to integrate oral health education into medical training, thus creating healthcare professionals proficient promoting oral health, has been encouraged [26]. In SA, educational programs can highlight the importance of oral hygiene for overall health, delivered through local mosques or local clinics with practical tips for overcoming physical limitations, such as using arthritis-friendly or electric toothbrushes. Incorporating oral health education into routine medical appointments at the SA MOH’s Primary Health Centers can reinforce these messages consistently.

While this study provides valuable insights into the toothbrushing habits of SA’s population and their associations with various sociodemographic and health factors, several limitations should be acknowledged. First, this study relies on self-reported data, which may introduce bias due to participants overreporting desirable behaviors, such as frequent toothbrushing, leading to social desirability bias. This limitation could skew our results, making it difficult to accurately assess true brushing behaviors in the population.

Additionally, while we observed significant associations, these relationships should not be interpreted as cause-and-effect. The cross-sectional nature of the study design captures data at a single point in time, making it impossible to determine the directionality of the associations or account for potential changes over time. This limitation implies that observed correlations may not accurately represent underlying causal relationships, highlighting the need for further longitudinal studies to explore these relationships.

Moreover, this study does not consider other oral hygiene practices, such as flossing, which could provide a more comprehensive understanding of oral health behaviors in the SA. We recognize that the limitations could alter our results and interpretations, and thus, we emphasize the importance of addressing these issues in future research.

## 5. Conclusions

This study reveals significant differences in toothbrushing frequency in Saudi Arabia, with only a small segment of the population meeting recommended oral hygiene practices. Socioeconomic status, gender, and regional differences significantly influence these behaviors. Addressing these disparities through targeted public health interventions can improve oral health outcomes and overall well-being of SA residents.

## Figures and Tables

**Table 1:** Brushing frequency and independent variables.

Variable Category	Independent Variable	Dependent Variable						
		Less than Once a Day		Once Daily		Twice or More Daily		* *p*-Value
		N	w%	N	w%	N	w%	
Total		25,954	57.3	11,788	26.9	7037	15.8	
Age group	5–14	6434	62.4	2558	25.3	1201	12.3	<0.001
	15–24	4950	56.0	2321	27.1	1495	16.9	
	25–34	5377	55.9	2681	27.4	1711	16.7	
	35–44	4299	55.0	1995	28.7	1252	16.3	
	45–54	2572	58.6	1194	24.8	718	16.6	
	55–64	1447	59.2	619	24.9	385	15.9	
	65+	875	51.4	420	30.9	275	17.7	
Gender	Male	13,776	58.6	5550	26.3	3168	15.0	<0.001
	Female	14,981	55.3	6927	27.7	4049	17.0	
Nationality	Saudi	26,173	57.7	11,159	25.7	6531	16.5	<0.001
	Non-Saudi	2584	56.5	1318	28.8	686	14.8	
Geographic Regions	North	2924	67.3	1059	24.9	364	7.8	<0.001
	South	6008	62.5	2035	23.9	1161	13.5	
	East	6110	63.6	2609	25.7	1025	10.7	
	West	8141	51.2	4331	30.9	2781	17.9	
	Central	5574	55.6	2443	25.1	1886	19.3	
Marital status of household head	Married	9268	57.1	4273	26.8	2684	16.1	0.023
	Not married	968	60.5	418	24.5	270	15.0	
Completed education level	Primary school education	4111	60.3	1698	26.7	851	13.0	<0.001
	Intermediate school education	7100	59.2	3037	25.5	1898	15.3	
	High school education	4748	55.8	2320	27.8	1415	16.4	
	Intermediate diploma	730	49.7	386	30.7	239	19.6	
	College or higher education	2506	50.5	1367	29.9	958	19.6	
Total Monthly Income	>22,901 Riyals	475	52.2	206	31.6	138	16.2	<0.001
	7700–22,900 Riyals	5700	55.6	2734	26.7	1789	17.7	
	3801–7699 Riyals	3286	57.3	1509	26.0	983	16.7	
	3800 Riyals or less	4030	60.2	1649	24.8	998	15.0	
Residence crowding	1 ≥ person per room	2071	60.2	845	24.6	524	15.1	<0.001
	1 to 2person per room	4950	57.1	2172	25.6	1447	17.3	
	2 < person per room	3875	56.1	1829	28.8	987	15.1	
Relevant medical conditions	Hypertension	1396	68.6	454	20.8	219	10.6	<0.001
	Diabetes	389	70.7	131	17.2	57	12.1	
	Other non-specified	3359	50.0	1938	29.1	1250	20.9	
History of physical accident	Yes	1387	55.0	618	26.9	405	18.1	0.003
	No	26,121	57.2	11,298	27.0	6511	15.8	
History of physical disability	Yes	462	57.4	187	23.8	88	18.8	0.024
	No	27,637	57.0	12,116	27.1	7040	15.9	
Smoking status	Smoker	1765	58.9	794	28.1	428	12.9	<0.001
	Non-smoker	21,508	56.6	9968	27.1	6114	16.3	
BMI	Underweight = <18.5	4230	62.2	1447	24.3	681	13.5	<0.001
	Normal weight = 18.5–24.9	8794	56.1	3848	27.4	2368	16.5	
	Overweight = 25–29.9	6244	55.0	3021	27.8	1925	17.1	
	Obesity = 30 or greater	3897	59.2	1778	26.7	911	14.1	
Household head health insurance	Yes	2840	60.5	1268	24.5	722	15.0	<0.001
	No	8500	55.7	3802	27.7	2333	16.6	
Dental health care availability in the last year	Available	6923	56.6	2944	26.1	1826	17.2	<0.001
	Not available	15,629	58.2	6538	26.9	3660	14.9	
	I don’t know	4114	55.7	1954	27.2	1097	17.1	
Dental visits frequency in the past year	Once	7321	65.1	2496	22.0	1344	12.9	<0.001
	More than once	7272	63.5	2627	23.9	1410	12.5	
	Not visited a dentist in the past year	9156	47.3	5400	32.3	3368	20.4	
	Never visited a dentist	2872	62.9	908	23.9	465	13.2	
	I do not know or do not remember	1217	48.4	731	32.0	449	19.6	
Reasons for last dental visit	Dental pain	12,954	64.9	4254	22.5	2281	12.6	<0.001
	Treatment and follow up	2466	66.4	901	23.6	397	10.1	
	Routine examination and treatment	1041	53.5	549	26.1	418	20.4	
	Don’t know	1302	46.1	878	32.3	533	21.6	
	Other reasons	221	52.4	116	27.0	120	20.6	
Regular source of dental care combined	Primary healthcare center	10,587	58.7	4590	26.5	2627	14.8	0.004
	Government hospital	1740	58.0	730	25.2	479	16.8	
	Privet clinic or hospital	3195	56.8	1464	26.8	951	16.3	
	Other	398	58.1	191	25.1	103	16.9	
Frequency of consuming sweets	I don’t eat at all	4875	65.7	1612	22.4	884	11.9	<0.001
	Many times per month	16,525	60.0	6577	24.8	3868	15.2	
	Many times per week	4362	45.1	2549	35.3	1457	19.6	
	At least once per day	2250	39.7	1604	38.2	910	22.2	
Frequency of drinking soft drinks	I don’t drink at all	9430	53.5	4452	28.5	2752	17.9	<0.001
	Many times per month	13,810	62.0	5245	24.5	2801	13.6	
	Many times per week	3599	52.6	1810	31.2	1046	16.2	
	At least once per day	1146	47.2	760	29.8	520	23.0	

* The analysis was performed using a Chi-square test.

**Table 2:** Multinomial regression analysis for relation between brushing frequency and independent variables.

Independent Variables		Once Daily vs. Less than Once Daily			Twice or More Daily vs. Less than Once Daily		
		OR	CI	* *p*-Value	OR	CI	* *p*-Value
Age	5–14	3.791	0.639–22.488	0.142	1.450	0.279–7.534	0.659
	15–24	9.248	1.585–53.962	**0.013**	4.377	0.898–21.340	0.068
	25–34	9.196	1.601–52.833	**0.013**	4.340	0.914–20.618	0.065
	35–44	7.970	1.384–45.894	**0.020**	2.788	0.574–13.538	0.203
	45–54	7.510	1.246–45.275	**0.028**	5.444	1.105–26.826	**0.037**
	55–64	8.285	1.288–53.288	**0.026**	2.887	0.514–16.212	0.228
	+65	reference					
Gender	Male	0.605	0.403–0.907	**0.015**	1.121	0.660–1.902	0.673
	Female	reference					
Nationality	Saudi	1.491	0.890–2.498	0.129	2.578	1.174–5.665	**0.018**
	Non-Saudi	reference					
Geographic Regions	North	1.409	0.608–3.264	0.424	1.098	0.255–4.732	0.900
	South	0.313	0.126–0.780	**0.013**	0.435	0.101–1.869	0.263
	East	1.724	0.940–3.161	0.078	1.258	0.479–3.299	0.641
	West	1.788	1.007–3.176	**0.047**	9.159	4.110–20.414	**<0.001**
	Central	reference					
Marital status for household head	Married	0.792	0.408–1.538	0.491	3.620	1.076–12.175	**0.038**
	Not married	reference					
completed education level	Primary school education	1.712	0.807–3.630	0.161	0.623	0.244–1.592	0.322
	Intermediate school education	0.961	0.504–1.835	0.905	0.445	0.200–0.988	**0.047**
	High school education	1.195	0.648–2.205	0.569	0.545	0.251–1.183	0.125
	Intermediate diploma	1.859	0.749–4.614	0.182	0.416	0.118–1.459	0.171
	College or higher education	reference					
Total Monthly Income	>22,901 Riyals	0.983	0.419–2.306	0.969	1.090	0.356–3.344	0.880
	7700–22,900 Riyals	0.915	0.540–1.551	0.741	0.866	0.409–1.833	0.707
	3801–7699 Riyals	0.790	0.457–1.366	0.399	0.809	0.377–1.736	0.586
	3800 Riyals or less	reference					
Residence crowding	≤1 person per room	0.555	0.332–0.927	**0.024**	1.409	0.745–2.667	0.291
	1–2 person per room	0.805	0.554–1.169	0.254	1.451	0.885–2.378	0.140
	>2 person per room	reference					
Relevant medical conditions	Hypertension	0.626	0.406–0.964	**0.033**	0.666	0.363–1.222	0.189
	Diabetes	0.452	0.221–0.921	**0.029**	0.435	0.151–1.254	0.123
	Other non-specified	reference					
History of physical accident	Yes	1.174	0.567–2.430	0.666	1.353	0.557–3.287	0.505
	No	reference					
History of physical disability	Yes	1.290	0.669–2.485	0.447	0.708	0.224–2.237	0.557
	No	reference					
smoking status	Yes	3.384	1.580–7.249	**0.002**	3.382	1.192–9.600	**0.022**
	No	reference					
BMI	Underweight = <18.5	2.782	1.258–6.153	**0.012**	0.963	0.321–2.884	0.946
	Normal weight = 18.5–24.9	1.771	1.075–2.916	**0.025**	0.900	0.476–1.701	0.745
	Overweight = 25–29.9	1.503	0.934–2.418	0.093	1.194	0.661–2.154	0.557
	Obesity = 30 or greater	reference					
household head health insurance	Yes	1.387	0.847–2.273	0.194	1.682	0.833–3.393	0.147
	No	reference					
Dental health care availability in the last year	Available	0.660	0.406–1.074	0.094	1.176	0.624–2.217	0.615
	Not available	0.568	0.345–0.934	**0.026**	0.772	0.389–1.532	0.459
	I don’t know	reference					
Dental visits frequency in the past year	Once	0.555	0.199–1.551	0.262	0.386	0.111–1.350	0.136
	More than once	0.979	0.358–2.675	0.967	0.566	0.166–1.929	0.363
	Not visited a dentist in the past year	0.645	0.226–1.838	0.412	0.287	0.077–1.072	0.063
	Never visited a dentist	1.630	0.398–6.684	0.497	1.181	0.206–6.779	0.852
	I don’t know or don’t remember	reference					
Reasons for last dental visit	Dental pain	0.590	0.175–1.990	0.395	0.803	0.120–5.356	0.821
	Treatment and follow up	0.545	0.147–2.019	0.364	0.371	0.044–3.106	0.360
	Routine examination and treatment	0.963	0.222–4.177	0.959	3.740	0.459–30.511	0.218
	Don’t know	0.232	0.052–1.028	0.054	1.226	0.144–10.459	0.852
	Other	reference					
Regular source of dental care	Primary healthcare center	1.333	0.522–3.403	0.548	0.407	0.125–1.328	0.136
	Government hospital	1.202	0.419–3.448	0.732	0.611	0.170–2.199	0.451
	Privet clinic or hospital	1.397	0.538–3.622	0.492	0.655	0.199–2.157	0.487
	Other	reference					
frequency of consuming sweets	I don’t eat at all	0.385	0.191–0.776	**0.008**	0.231	0.081–0.653	**0.006**
	Many times per month	0.383	0.209–0.704	**0.002**	0.595	0.275–1.288	0.188
	Many times per week	0.408	0.211–0.790	**0.008**	1.367	0.616–3.036	0.442
	At least once per day	reference					
frequency of drinking soft drinks	I don’t drink at all	2.281	0.971–5.354	0.058	1.672	0.555–5.039	0.361
	Many times per month	1.869	0.813–4.294	0.141	1.037	0.349–3.080	0.948
	Many times per week	2.391	1.001–5.711	0.050	3.331	1.131–9.811	**0.029**
	At least once per day	reference					

* Multinomial logistic regression analysis. Bold values denote statistical significance at the *p* < 0.05 level.

## Data Availability

The data were available from the office of Directorate of Primary Health Care Centers (MOH headquarters, Riyadh, Saudi Arabia).
